# Risk-adapted HLA delisting and imlifidase-enabled deceased-donor kidney transplantation in highly sensitized kidney transplant candidates: a German expert consensus report

**DOI:** 10.3389/fimmu.2026.1846251

**Published:** 2026-06-09

**Authors:** Stefan Reuter, Louise Benning, Andrea Dick, Lisa-Marie Dilz, Gunilla Einecke, Florian Emmerich, Florian Grahammer, Rebecca Großmann, Wilfried Gwinner, Fabian Halleck, Michael Hallensleben, Falko M. Heinemann, Bernd Jänigen, Dennis Kannenkeril, Teresa Kauke, Reinhard Kelsch, Stephan Kemmner, Malte Andreas Kluger, Jan Kowald, Andreas Kribben, Claudia Lehmann, Monika Lindemann, Giancarlo Maccagno, Christian Morath, Anja Mühlfeld, Lien Pham, Lutz Renders, Stefan Schaub, Sabine Scherer, Christian Seidl, Bernd Spriewald, Dirk Stippel, Julian Stumpf, Thuong Hien Tran, Rolf Weimer, Benjamin Wilde, Daniel Zecher, Malte Ziemann, Svitlana Ziganshyna, Nils Lachmann, Klemens Budde

**Affiliations:** 1 Department of Medicine D, Transplant Nephrology, University Hospital Münster, Münster, Germany; 2 Deutsche Transplantationsgesellschaft (DTG) Kidney Commission, Regensburg, Germany; 3 Department of Nephrology, Heidelberg University Hospital, Heidelberg, Germany; 4 Department of Transfusion Medicine, Cell Therapeutics and Hemostaseology, Laboratory for Immunogenetics and Molecular Diagnostics, Ludwig-Maximilians-Universität (LMU) University Hospital Munich, Munich, Germany; 5 Institute of Transfusion Medicine, Laboratory for Transplantation Immunology, Leipzig University Hospital, Leipzig, Germany; 6 Department of Nephrology and Rheumatology, Section of Kidney Transplantation, University Medical Center Göttingen, Göttingen, Germany; 7 Deutsche Transplantationsgesellschaft (DTG) Immunology Commission, Regensburg, Germany; 8 Deutsche Gesellschaft für Immungenetik (DGI) Organ Transplantation Commission, Regensburg, Germany; 9 Institute for Transfusion Medicine and Gene Therapy, Freiburg University Medical Center, Faculty of Medicine, University of Freiburg, Freiburg, Germany; 10 III. Department of Medicine, Nephrology/Rheumatology/Endocrinology, University Medical Center Hamburg-Eppendorf (UKE), Hamburg, Germany; 11 Hamburg Center for Kidney Health (HCKH), University Medical Center Hamburg-Eppendorf (UKE), Hamburg, Germany; 12 Institute of Transfusion Medicine, Stem Cell Donor Registry Leipzig, Leipzig University Hospital, Leipzig, Germany; 13 Department of Nephrology, Hannover Medical School, Hannover, Germany; 14 Department of Nephrology and Medical Intensive Care, Charité – Universitätsmedizin Berlin, Berlin, Germany; 15 Institute for Transfusion Medicine and Transplant Engineering, Hannover Medical School, Hannover, Germany; 16 Institute for Transfusion Medicine, Transplantation Diagnostics, University Hospital Essen, Essen, Germany; 17 Department of General and Visceral Surgery, Section of Transplantation Surgery, Medical Center – University of Freiburg, Freiburg, Germany; 18 Department of Medicine 4, Nephrology and Hypertension, University Hospital Erlangen, Erlangen, Germany; 19 Department of Thoracic Surgery, Lung Transplantation and Transplant Immunology, Ludwig-Maximilians-Universität (LMU) University Hospital Munich, Munich, Germany; 20 Institute of Transfusion Medicine and Cell Therapy, Laboratory for Immunogenetics/Human Leukocyte Antigen (HLA), University Hospital Münster, Münster, Germany; 21 Transplant Center, LMU University Hospital Munich, Munich, Germany; 22 Division of Nephrology, Department of Endocrinology, Nephrology, and Rheumatology, University of Leipzig Medical Center, Leipzig, Germany; 23 University Hospital Essen, Essen, Germany; 24 Institute of Transfusion Medicine, Transplant Immunology, University Hospital Hamburg, Hamburg, Germany; 25 Institute of Transfusion Medicine, Transplant Immunology, University Medical Center Mainz, Mainz, Germany; 26 Department of Nephrology and Hypertension, Nuremberg Hospital and Paracelsus Medical Private University, Nuremberg, Germany; 27 Division of Nephrology and Immunology, Uniklinik Rheinisch-Westfälische Technische Hochschule (RWTH) Aachen, Aachen, Germany; 28 Institute for Immunology, Transplantation Immunology, University Hospital Heidelberg, Heidelberg, Germany; 29 Department of Nephrology, TUM University Hospital Klinikum rechts der Isar, Technical University of Munich, Munich, Germany; 30 Clinic for Nephrology and Transplantation Immunology, University Hospital Basel, Basel, Switzerland; 31 Institute of Transfusion Medicine and Immunohematology, University Hospital Frankfurt, Frankfurt am Main, Germany; 32 Laboratory of Immunogenetics (HLA Laboratory), Departments of Medicine 3 and 5, University Hospital Erlangen, Erlangen, Germany; 33 Department of General, Visceral, Tumor and Transplantation Surgery, University Hospital Cologne, Cologne, Germany; 34 Division of Nephrology, Department of Internal Medicine 3, University Hospital Carl Gustav Carus at the Technische Universität Dresden, Dresden, Germany; 35 Department of Internal Medicine, Nephrology and Renal Transplantation, University Hospital of Giessen and Marburg, Giessen, Germany; 36 Department of Infectious Diseases and Nephrology, University Hospital Essen, University of Duisburg-Essen, Essen, Germany; 37 Department of Nephrology, University Hospital Regensburg, Regensburg, Germany; 38 Institute of Transfusion Medicine, University Hospital Schleswig-Holstein, Lübeck, Germany; 39 Organ Donation Coordinator Unit, University of Leipzig Medical Center, Leipzig, Germany; 40 Institute of Transfusion Medicine, Tissue Typing/Human Leukocyte Antigen (HLA), Charité – Universitätsmedizin Berlin, Berlin, Germany

**Keywords:** delisting, highly sensitized patient, HLA incompatibility, imlifidase, kidney transplantation

## Abstract

Kidney transplantation is the preferred treatment for suitable patients with end-stage renal disease; however, access to transplantation declines dramatically with increasing HLA sensitization. While the acceptable mismatch (AM) program by Eurotransplant improves transplantability for highly sensitized candidates, a clinically relevant subgroup with extremely low donor frequency or ineligible for AM remains disadvantaged. In carefully selected cases, the controlled delisting of unacceptable HLA antigens and the use of peri-transplant desensitization (e.g., imlifidase) may enable transplantation. In order to provide better guidance, this German expert consensus report was compiled by the Kidney and Immunology Commissions of the German Transplantation Society and the Organ Transplantation Commission of the German Society for Immunogenetics. Within the German legal framework of urgency and chances for success, the report proposes a practical guide for candidate selection, multidisciplinary governance, risk-adapted HLA delisting, assessment of organ offers, use of imlifidase, perioperative immunosuppression, prophylaxis for infection, post-transplant monitoring, and management of antibody-mediated rejection. These recommendations are intended for experienced transplant centers and aim to balance transplant opportunity against immunological risk in highly sensitized kidney transplant candidates. The primary scope of this article is highly sensitized adult wait-listed candidates considered for deceased-donor kidney transplantation after compatibility-preserving pathways have been exhausted or are unlikely to succeed; HLA-incompatible living-donor transplantation with imlifidase is addressed separately as a potential off-label scenario for selected highly sensitized patients.

## Introduction

Between 2011 and 2019, the proportion of highly sensitized (HS) kidney transplant candidates with a calculated, virtual HLA panel reactivity (vPRA) of at least 85% more than doubled in the Eurotransplant (ET) region—from 2.0% to 5.6% of all listed patients (1). Since the degree of HLA sensitization correlates with the probability of transplantation in the Eurotransplant Kidney Allocation System (ETKAS) and the Eurotransplant Senior Program (ESP), HS patients are significantly disadvantaged in these programs (2, 3). However, their chances improve significantly if they meet the criteria for inclusion in the acceptable mismatch (AM) program (1, 3). Below a donor frequency of 0.1%, however, the probability of transplantation decreases dramatically. Even within the AM program, the annual probability of transplantation for this group of patients is less than 2%, while dialysis-related mortality is significantly higher (2, 4). For clinically suitable patients, desensitization followed by HLA-incompatible transplantation may offer a survival advantage over those remaining on the waiting list (5, 6).

Current data by ET underscore this imbalance. As of 1 August 2025, 252 German patients with a vPRA of at least 99.5% were on the active waiting list. Of those patients, only 124 were listed in the AM program, and 52 had waited ≥9 years for a transplant. In the 8 years prior to this date (2017–2025), only 24 German patients with a vPRA of at least 99.5% received a kidney transplant, 20 of whom received one via AM (source: personal communication with H. de Ferrante, ET). A recent waiting list analysis of four German transplant centers also showed that >10% of active patients, who are not listed in the AM program, have a vPRA >95% (7).

To improve the chances of this patient group receiving a suitable transplant offer, several strategies have been established worldwide: prioritization within allocation systems (such as the AM program), kidney paired donation programs, and sequential delisting usually combined with various desensitization protocols (1, 8–14). Recently, ET has explicitly integrated the option of desensitization into its allocation programs (formal recommendation R-KAC01.25).

The immunoglobulin G (IgG) endopeptidase imlifidase (Idefirix^®^, Hansa Biopharma, Lund, Sweden) cleaves human IgG at the hinge region in two steps, thereby reducing the HLA antibody (HLAab) load below the detection limit within 60–240 min and converting a previously positive crossmatch (XM), which is a contraindication for kidney transplantation, into a negative result (15, 16). In a phase II study, a dose of 0.25–0.50 mg/kg body weight resulted in successful XM conversion in 89.5% of patients (16). This procedure creates a 5–8-day window of opportunity for transplantation before the HLAabs return back to measurable levels, thereby overcoming the previously existing immunological HLA barrier. Long-term follow-up showed a 5-year patient survival rate of 90% and a death-adjusted 5-year graft survival rate of 82% (17). However, approximately 40% of patients develop clinically manifest antibody-mediated rejection (AMR), in addition to potential subclinical AMRs that have not yet been well characterized in protocol biopsies (16, 19).

Imlifidase is the first and currently only approved therapy for the specific desensitization of adult HS kidney transplant patients receiving a deceased donor. Following its approval by the European Medicines Agency (EMA) in August 2020, a French recommendation for the use of imlifidase was proposed in 2023 (19). In 2025, the Belgian Transplantation Society published a comprehensive, operational protocol for HS patients (20). At the same time, an international Delphi consensus developed additional best practice recommendations (21).

This article is a joint expert recommendation by members of the “Kidney” and “Immunology” commissions of the German Transplantation Society (DTG), the “Organ Transplantation” commission of the German Society for Immunogenetics (DGI), and experts from German transplant centers. It is based on the previous 2022 German consensus recommendations for defining NAHAs (non-acceptable HLA antigens) and integrates the specific legal aspects and current guidelines for Germany with the international literature (11). The recommended NAHA treatment strategy in this report is primarily based on the experience of six successful imlifidase-assisted kidney transplants at the Charité-Universitätsmedizin Berlin Transplantation Center (22).

## The acceptable mismatch program and the role of imlifidase

ET established its AM program over 30 years ago to provide HS a better chance of an immunologically compatible donor organ, usually without desensitization, despite broad HLA sensitization (1). Currently, transplant-eligible patients listed in ETKAS who have waited at least 2 years, have at least one measurable cytotoxic HLAab, and have a vPRA ≥85% (verified by the ET reference laboratory ETRL using representative serum samples) are eligible for the AM program. In the future, admission will be based on an ET donor frequency of <2% (based on NAHAs and blood group identity) (ET Manual, R-TTAC01.22a/b). In the AM program, HLA antigens are defined as acceptable mismatches for which there are no relevant HLAabs, either historically or currently. These antigens are treated as putative self-antigens in the allocation, which significantly expands the potential donor pool (1). Candidates listed in the AM program receive the highest priority in the allocation, and >80% undergo transplantation within 3 years. To date, the program has enabled >1,700 HS patients to be transplanted with similar graft survival rates to those of significantly less sensitized recipients (23). Limitations primarily exist in rare HLA phenotypes/haplotypes or in candidates who do not meet the AM inclusion criteria (1, 2).

### Supplementary desensitization using imlifidase

Since 2023, ET has opened the option of placing AM patients on a separate desensitization list (the AM desensitization program) alongside the AM program. This allows the delisting of unacceptables in patients with long waiting times on the AM list in order to allow allocation for organs with delisted unacceptables, giving those who have not received an organ despite AM prioritization a chance for transplantation (ET Manual). The goal is to evaluate novel desensitization strategies in a small group of patients within a well-defined framework including the use of imlifidase. To date, 26 patients have been evaluated in this program. Of those, 20 patients were ultimately registered, and 10 have already been transplanted as of 25 June 2025, including two in Germany. Initial analysis showed that only some suitable AM patients and qualified transplant centers took advantage of the desensitization offer. However, the program is currently suspended in Germany following intervention by the German Medical Association (Bundesärztekammer, BÄK) because the legal requirements necessitate a change in guidelines. Nevertheless, imlifidase remains an option outside the AM program for center-based protocols in cases of positive virtual crossmatch (vXM), but without AM prioritization. The organ must be acquired via ETKAS (see below) or through living donation.

### Alternatives

For HS patients who cannot be included in the AM program or for whom no adequate offers are generated despite AM status, the following options are currently considered in Germany: controlled delisting of unacceptables, supported by conventional desensitization regimens with intensified immunosuppression post-transplant, or potentially through the use of imlifidase. An interdisciplinary team and the transplant conference should carry out the delisting of unacceptables in a risk-adapted manner, analogous to the proposed delisting in the AM desensitization program. In case of an organ offer with a positive virtual XM, this process could be combined with the use of imlifidase. Controlled delisting is particularly indicated for sensitized patients with life-threatening dialysis-related complications, such as recurrent access problems (e.g., shunt thrombosis) and/or peritoneal dialysis failure. In candidates with a willing but immunologically incompatible living donor, kidney paired donation and altruistic donor-initiated living-donor chains should be evaluated first where legally and operationally available, as it may avoid or reduce desensitization (24, 25). In selected very highly sensitized candidates, paired donation may be combined with tailored desensitization as done in other countries (24–26). Imlifidase-assisted living-donor transplantation remains off-label and should be reserved for selected cases after MDT review, patient counseling, and reimbursement clarification (see below). Finally, novel experimental cell therapies such as CD19 CAR T cells or bispecific T-cell-engaging antibodies targeting plasma cells may change the field of desensitization in the future and may be a potential alternative for highly selected patients willing to participate in clinical trials (27–29). A comparative overview of compatibility-preserving and access-enabling strategies for highly sensitized candidates is provided in **Table 1**. Currently, many recommendations are based on expert opinion and lack high-level scientific evidence. However, registries and ideally prospective clinical trials are key to further advance the field. Systematic subgroup analyses (immunological, clinical, and sociodemographic) and registry participation are necessary to optimize results and continuously improve patient selection, delisting criteria for unacceptables, post-transplant monitoring, and concomitant immunosuppression.

**Table 1:** Compatibility-preserving and access-enabling strategies for highly sensitized kidney transplant candidates.

Strategy	Main setting	Potential advantage	Main limitation	Role in this consensus
Compatible allocation/AM program	Wait-listed HS candidates	Avoids therapeutic barrier crossing; best immunological starting point	Not sufficient for very low donor frequency, rare HLA phenotypes, or poor matchability	Preferred pathway whenever realistic
Kidney paired donation/compatible living-donor pathway	Incompatible living donor available	May avoid or reduce desensitization	Depends on program size, legal framework, and match probability	Should be evaluated before off-label imlifidase in living donation
Conventional desensitization	Potential for planned living-donor or controlled settings	Scheduled treatment and iterative antibody monitoring	Limited efficacy, Antibody rebound, frequent AMR, high treatment burden	Selective option after compatible pathways fail
Risk-adapted delisting	Very low donor frequency, selected HLA antibodies	Expands the virtual compatible donor pool	Requires expert HLA interpretation; AMR risk persists	Core component of the proposed German pathway
Imlifidase-enabled deceased-donor transplantation	Positive vXM/physical XM against available deceased donor	Rapid IgG cleavage and short transplant window	Early DSA rebound, frequent AMR, increased costs and logistics	Highly selected in-label access-enabling strategy
Imlifidase-enabled living-donor transplantation	Positive vXM/physical XM against available living donor	Optimal donor, scheduled treatment for IgG cleavage and potential for additional pretransplant activities	Off-label, early DSA rebound, frequent AMR, increased costs and logistics	Highly selected off-label access-enabling strategy
Investigational desensitization with novel therapies such as CAR-T cells or bispecific T-cell engager	Availability of investigational desensitization	Mechanistically promising plasma-cell/immune-cell targeting therapies to reduce antibody rebound and AMR	Off-label; highly experimental No long-term safety and outcome data	Investigational for highly selected patients

## Practical implementation: infrastructure and team

- Multidisciplinarity: The complex selection, preparation, care, and treatment of patients requires an effective multidisciplinary team (MDT) with specific experience in transplant immunology, kidney transplantation, and AMR treatment. Due to the complexity and low number of cases in Germany, expertise should be concentrated in a few centers initially.
- Core imlifidase team: Should be available 24/7 and proficient in the imlifidase protocol:
· nephrology: responsible for waiting list management and follow-up care,
· experienced transplant surgery, including retransplantations,
· HLA laboratory with proven expertise,
· pathology: responsible for biopsy evaluation, and
· transplant coordination team.
- Pharmacy: Imlifidase is available 24/7.
- *Case discussion in the transplant conference*: All potential candidates are discussed in detail in advance by the MDT and then confirmed in the interdisciplinary transplant conference, where the procedure is documented. A risk-adapted treatment strategy and organ acceptance criteria are defined and documented. Details on anti-infective or AMR adjunctive therapy are adapted to the respective center’s practice on a case-by-case basis.
- Information: The patient is informed of the various options, including the delisting strategy, and is given comprehensive information about the possible risks, side effects, and chances of success.
- Implementation: Only after the patient has provided written consent and the interdisciplinary transplant conference has decided, considering the urgency and chances for success, will the delisting strategy and specified treatment protocol be implemented.
- Transplant immunology: The timing of sample collection to determine HLA sensitization before and monitoring of donor-specific HLAab (DSA) after transplantation is generally based on the clinical judgment of the treating physicians. However, they must comply with the German Medical Authorities’ (Bundesärztkammer) guidelines, the standards of the European Federation for Immunogenetics (EFI), and the requirements of Chapter 10 (Histocompatibility) of the ET Manual. Prior to transplantation, further analyses are necessary to characterize HLAabs in detail to assess their potential pathogenicity. This information helps to predict the reduction of HLAabs under imlifidase immediately prior to transplantation and the antibody rebound after transplantation. To this end, single antigen bead (SAB) assays are recommended for detecting IgG antibodies. If necessary, these assays might be supplemented by binding assays of complement components C1q, C3d, or C4d, as well as serum dilutions. Measures should be taken to minimize the effect of complement interference (30, 31).
- Kidney biopsies: Ideally, biopsy results should be available within two working days. In most centers, it is not possible to obtain results on weekends for organizational reasons. In urgent cases, frozen section analysis is recommended to bridge the time until the final histological results are available. If acute rejection is suspected based on clinical findings, treatment should be initiated immediately, regardless of whether the biopsy results are available.

## Patient selection

The recommended inclusion and exclusion criteria for patients to receive imlifidase treatment after delisting vary in the literature. **Table 2** provides an overview of various European recommendations.

**Table 2:** Inclusion and exclusion criteria (Belgium, France, Delphi, Germany).

Criteria	Germany	Belgium	France	Delphi consensus
Age	≤65 years	≤65 years	≤65 years	Not defined
v/cPRA level	≥95%	≥85%	≥98%	≥98%
Waiting time	≥8 years	≥36 months (AM) or ≥48 months (ETKAS)	Not defined (mostly >8 years)	Shortening possible in cases of vital indication (MDT)
Previous transplant	No specification	No specification	>2 = exclusion	No specification
On dialysis	Yes	Yes	Yes	Yes
Crossmatch	Virtually positive, no physical XM before imlifidase, negative T-CDC before KTx	Virtually positive, negative T-CDC before KTx	Virtually positive, no physical XM before imlifidase	Virtually or physically positive, negative T-CDC before Tx
Indication for imlifidase (vXM criterion)	Recommendation: cumulative MFI of HLA-A, HLA-B, HLA-DRB1, HLA-DQA1, and HLA-DQB1 DSA >10,000 MFI with at least one DSA >5,000 MFI using One Lambda’s SAB	Cumulative MFI of all HLA-A, HLA-B, HLA-DRB1, HLA-DQA1, and HLA-DQB1 DSA >6,000 MFI with at least one DSA >2,000 MFI using Werfen’s SAB	MFI of the immunodominant DSA >6,000 (excluded HLA-C and HLA-DP) by One Lambda’s SAB	n/a
MDT approval	Necessary	Necessary	Not necessary	Necessary
Patient consent	Necessary	Necessary	Necessary	Necessary
Infection status	No active infection	No active infection	No active infection	No active infection
Further exclusion criteria	Severe coagulopathy, lack of infrastructure frailty	Severe coagulopathy, lack of infrastructure	Severe coagulopathy, lack of infrastructure	Clinical decision MDT
Delisting strategy	Individually	Individually, MFI-based, MDT-defined	Individually, MFI-based, MDT-defined	Individually, MFI-based, MDT-defined (with local options)
Registry/study	Recommended	Recommended	Recommended	Recommended

MDT, multidisciplinary transplantation team; SAB, single antigen bead assay.

### Exclusion criteria for patient selection

#### Relative exclusion criteria

- Language barrier
- Permanent anticoagulation (need for kidney biopsies)

#### Absolute exclusion criteria

- Frailty/operability: Not suitable if the requirements described under “Patient selection: operability/frailty” are not met.
- Active infection or lack of basic immunizations (including pneumococcal and meningococcal vaccines), as C5 blockade may be necessary.
- Severe coagulopathy
- Lack of infrastructure at the center (see above).
- Contraindications according to the imlifidase smPC.

### Inclusion criteria

1. HLA sensitization level: vPRA ≥95% (or donor frequency <2%), based on unacceptables. In these cases, the likelihood of receiving a compatible organ decreases dramatically (2, 3). All alternative options (i.e., HLA-compatible living donation, inclusion in the AM program, delisting under conventional desensitization) must be exhausted beforehand.

2. Waiting time: The individual waiting time should generally exceed the median for blood group-identical patients in ETKAS (i.e., a total of 8–10 years) and/or exceed 3 years in the AM program without receiving a compatible organ offer, as the likelihood of receiving a suitable organ offer decreases significantly with waiting times exceeding 3 years in AM.

3. Exceptions: i) In cases of medical urgency due to a lack of or foreseeable exhaustion of dialysis access options, ii) in cases of very high HLA sensitization with vPRA>99.5% and very low donor frequency ≤0.1% in the AM program, or iii) in cases where a living donor is available, the abovementioned prerequisite waiting time may be waived for good reason.

4. Crossmatch: The following aspects must be considered with regard to crossmatch requirements:

4.1. Allocation: The legal prerequisite for allocating a postmortem donor organ via ETKAS is a negative vXM. Unacceptables are delisted in anticipation of the complete elimination of IgG antibodies within 2 to 4 h after administration of imlifidase.

4.2 In-label: After allocation and before transplantation, a positive virtual or physical crossmatch must be given and documented (prerequisite for administering imlifidase). Subsequently, the crossmatch must convert to negative and must be documented.

4.3 Transplantation: Immediately prior to transplantation and after desensitization with imlifidase, a negative prospective CDC-XM must be performed at the transplantation center at least at the sensitivity level required by the German Medical Authorities’ guidelines (German Medical Authorities’ guidelines, “Recipient Protection” and “Kidney Transplantation”) and Eurotransplant histocompatibility requirements. FC-XM may additionally be performed according to local center practice and should be integrated into the final MDT risk assessment, particularly in immunologically complex cases Note: In a French registry cohort (French ISKIA registry, unpublished), 86% of previously CDC-positive crossmatches and 95% of FC-positive crossmatches converted to negative results within 4 to 6 h after imlifidase administration. Persistent positivity may be due to neutralizing antibodies against imlifidase from previous exposure to *Streptococcus pyogenes*, which may be partially overcome by administering a second dose.

5. Age/frailty: The intensified treatment regimen, which includes managing potential AMR episodes, is generally not suitable for patients with multiple chronic conditions or who are frail. While it is not possible to define a rigid age limit, the peri- and postoperative risk typically increases around the age of 60–65 due to the frequent presence of comorbidities. Therefore, medical, psychological, and physical suitability for surgery, potential complications, and intensive immunosuppression must be assessed on an individual basis and in an interdisciplinary manner. We recommend a standardized frailty assessment (e.g., the Fried phenotype). A Fried score of 3 or higher is associated with an increased perioperative and postoperative risk, as well as more complications after kidney transplantation (32, 33). In cases of Fried ≥3, imlifidase-based desensitization should generally not be performed. Prehabilitation may be considered in individual cases. This requires comprehensive information about the benefits and risks, as well as a clear desire for transplantation on the part of the patient.

6. Approval by the interdisciplinary transplant conference.

7. Written patient information and consent should be obtained early on, after the decision to delist in the MDT is made, and again immediately before transplantation.

## Delisting strategy

The goal of risk-stratified delisting is to increase the probability of allocation for HS patients without increased risks associated with immunologically inadequate donor organs or significantly endangering long-term success after transplantation. Delisting decisions are made on an individual basis, considering the patient’s historical and current immunological profile, clinical history, and MDT assessment. A comprehensive review of the immunization history is essential in this regard. The goal is to increase donor frequency, stratified by risk, to over 2%, if possible. Historical HLAabs or HLAabs with repeatedly low MFI, which are only detectable by the SAB assay, should be delisted first. Conversely, repeated mismatches with detectable antibodies from previous transplants or pregnancies should remain unacceptable, especially if they are complement-binding, as they could lead to a positive CDC-XM. This strategy requires a comprehensive review of the patient’s immunization history, including HLA typing of potential child’s father and retesting of previous transplants.

### Overview of the basic principles for delisting

1. Individual risk–benefit assessment: There is no clear upper limit for NAHAs to be delisted. The responsible MDT has the discretion to make this decision.
2. Critical review of current unacceptables: Critically examine the reasons for determining the current unacceptables for each HLA characteristic. If necessary, implausible unacceptables should be delisted as an initial step (11).
3. In case of doubt, it is recommended that you consult with other transplant immunologists who are experts in delisting unacceptables.
4. Each additional delisting step should be discussed in the MDT, and patients should be reeducated/informed about the increased risks.
5. A moderate increase in donor frequency through selective delisting should be the goal. Success in delisting unacceptables should be confirmed by a change in donor frequency. The goal is to increase donor frequency to over 2%.

### Implementation of delisting in ENISnext

The allocation-effective delisting of NAHAs takes place in the Eurotransplant Network Information System ENISnext and must be documented there.

#### Recommended delisting algorithm

**Figure 1:**
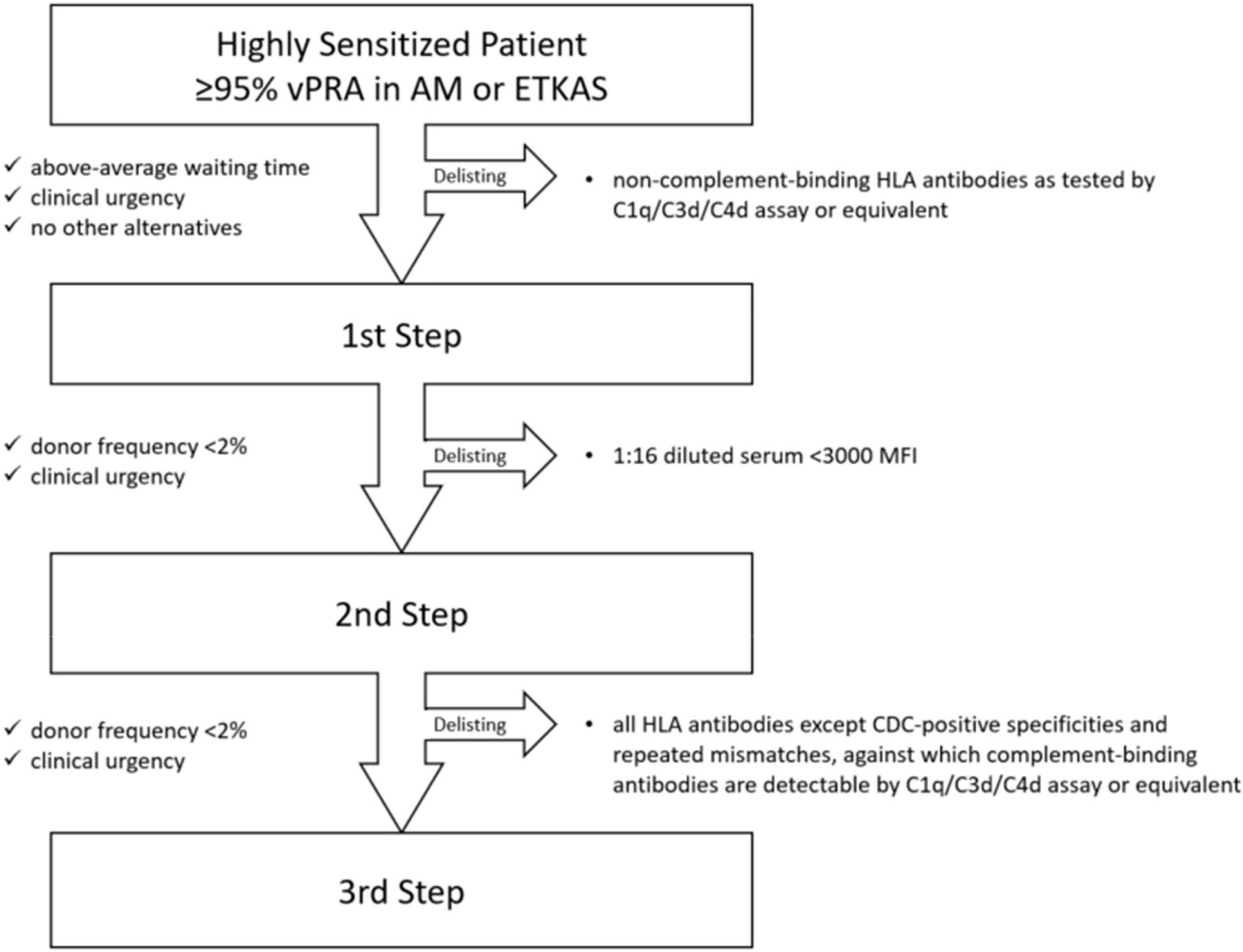
Risk-adapted stepwise delisting strategy for non-acceptable HLA antigens.

(Step-by-step escalation, see also **Figure 1**).

Step 1: Delisting of unacceptables with proven, plausible antibodies that are currently either undetectable or detectable as non-complement-binding by a C1q/C3d/C4d assay. Alternatively, the potential complement-binding capacity of HLAabs might be estimated to a limited extent based on the SAB assay results and the mean fluorescence intensity (MFI) of the standard IgG assay (34–36).

Step 2: Delisting of IgG antibodies <3,000 MFI SAB assay in 1:16 diluted current serum (serum sample from the last quarter).

Stage 3: Delisting of all unacceptables except those detectable by lymphocytotoxicity test (LCT/CDC) and antibodies from repeated incompatibilities that are detectable as complement-binding by a C1q/C3d/C4d assay or have MFI equivalent to the complement-binding assays in the standard IgG assay from undiluted serum.

##### HLA locus prioritization

HLA-A, HLA-B, HLA-DRB1, HLA-DQA1, HLA-DQB1: must be considered according to the above scheme.

HLA-C, HLA-DRB3/4/5, HLA-DPB1, HLA -DPA1: consideration optional, as the literature evidence is limited.

Consider the following additional risk parameters:

- Level of cumulative MFI (see below), particularly for epitopes Bw4 and Bw6.
- Historical HLA antibodies may also need to be taken into account.
- Repeated mismatches (RMM) in the HLA-DQA/DQB complex with significantly detectable HLA antibodies (i.e., MFI >5,000) should be avoided if possible and should not be delisted (37).
- Antibodies with shared eplets to HLA antigens from previous events.
- RMM without detectable HLAabs: RMM in HLA class I are more acceptable. RMM in HLA class II should only be accepted in individual cases under close monitoring (37).
- Delisting of RMM with antibodies is possible in principle, but there is a high risk of early rebound.

##### Non-delistable antigens (exclusion criteria)

Antigens against which IgG antibodies are directed, which are detectable in the current serum by means of LCT with the addition of dithiothreitol (DTT) as cytotoxic, may not be delisted (by German Law) and Eurotransplant Manual Chapter 10 (Histocompatibility), as they are highly likely to cause a positive CDC-XM and are therefore considered a contraindication for kidney transplantation.

In general, this recommendation establishes three levels (1–3), each with a different delisting profile. Delisting typically starts at level 1 and may escalate to levels 2 and 3, if necessary. Consider switching to a higher risk level if either of the following conditions is met: i) The calculated donor frequency is less than 2%. ii) There is clinical urgency, and the previously defined time interval (e.g., 6 months) has passed without adequate organ offers. Information about patients adapted to the higher risk level must be provided.

### Requirements at the time of transplantation

1. Negative virtual crossmatch (vXM) in the context of allocation by ET: This may only be possible if unacceptables are delisted.

2. Positive virtual transplant crossmatch at the transplant center when an organ is available (taking into account the deleted unacceptables as part of the delisting): The last serum test using the SAB assay should not be older than 3 months, and no potential immunization event should have occurred since then. The center-specific definition of a positive vXM must be agreed upon and defined in advance in the MDT. For example, the cumulative MFI of all mismatches (including or excluding HLA-C, HLA-DRB3/4/5, HLA-DPB1, or HLA-DPA1, depending on center policy) can be calculated. In general, the indication for using imlifidase or other desensitization strategies should be given by the positive virtual transplant crossmatch, taking into account the NAHAs deleted by delisting, if the cumulative MFI in undiluted serum (normalized) of the DSA is ≥10,000 MFI with at least one DSA >5,000 MFI against HLA-A, HLA-B, HLA-DRB1, HLA-DQA1, or HLA-DQB1.

Additionally, in the case of treatment with imlifidase.

3. Negative virtual transplant crossmatch from serum 4 h after imlifidase administration. A standard IgG SAB assay prior to transplantation from serum obtained at least 4 h after imlifidase administration and using a suitable methodology to minimize complement interference should confirm the conversion of the positive virtual transplant crossmatch from step 2.

4. Negative prospective CDC/FC-XM obtained from serum 4 h after imlifidase administration. The final transplant crossmatch prior to transplantation must be negative in accordance with the German Medical Authorities guidelines and ET Manual Chapter 10 Histocompatibility, using at least isolated T lymphocytes or unseparated cells and at least at the sensitivity level of the LCT/CDC using DTT.

### Donor criteria and logistics

When accepting an organ, it must be clarified whether imlifidase is indicated for the specific offer (i.e., whether vXM is positive).

Cold ischemic time (CIT) should be kept as short as possible, as it is associated with delayed graft function (DGF) and graft survival. The effect is more pronounced in older donors and sensitized recipients (38–40). Recommendation: Aim for CIS of 12–15 h or less; exceed 18–20 h only in exceptional cases where there is a favorable donor profile. Marginal donor organs should be avoided. In imlifidase protocols, delays related to the procedure due to the duration of action of imlifidase and the additional crossmatch must be taken into account. The processes should therefore be planned so that the CIT falls within the target range (18, 30). We recommend obtaining the surgeon’s approval for the organ before administering imlifidase, or at least carrying out an initial assessment.

### Imlifidase protocol

**Figure 2:**
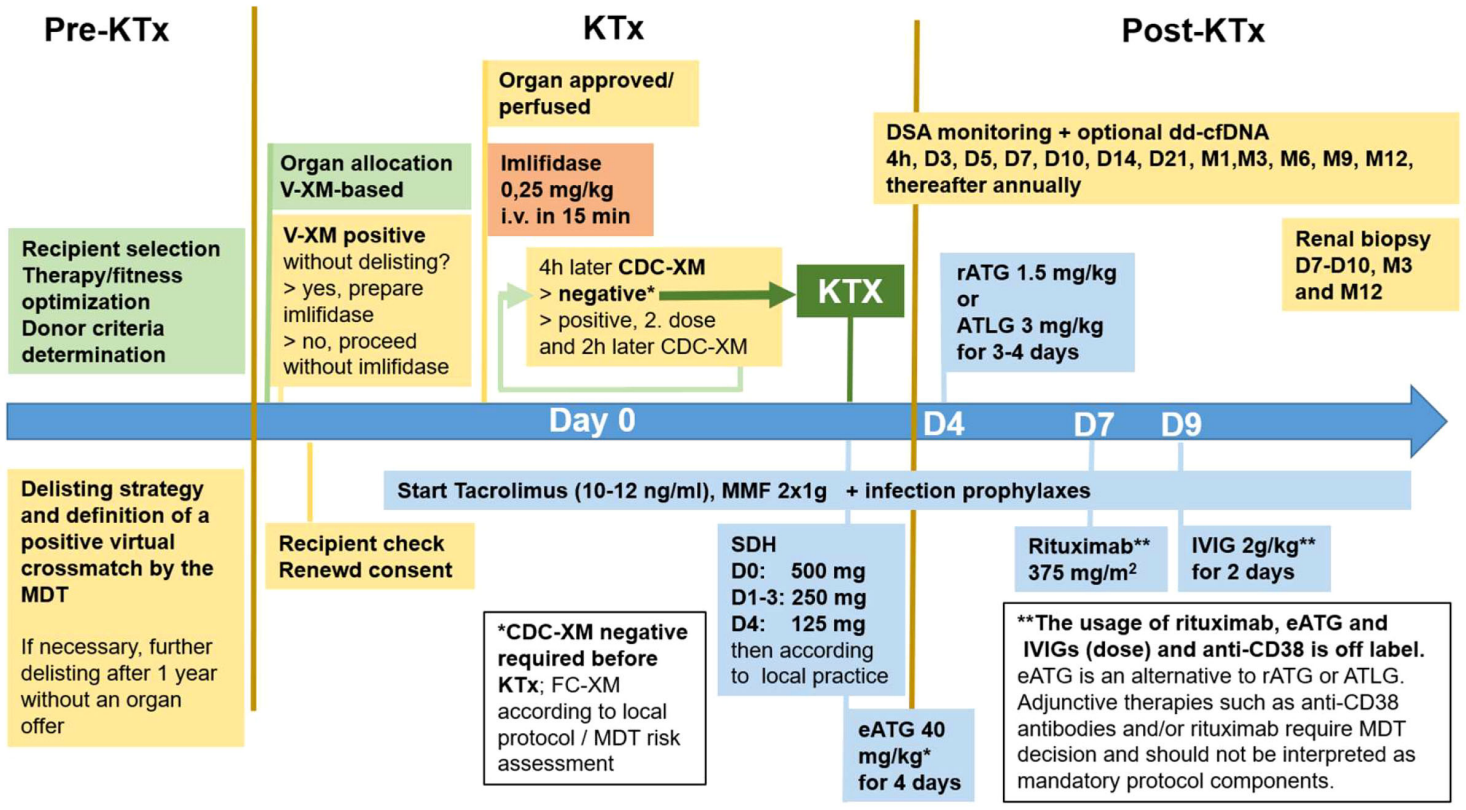
Timeline of an imlifidase-assisted kidney transplant in highly sensitized patients. The diagram shows recipient selection, vXM-based allocation, use of imlifidase in the event of a positive virtual crossmatch, perioperative crossmatch checks, and the post-transplant immune and monitoring regimen, including DSA monitoring. Off-label treatment options are indicated.

For the overall procedure, see also **Figure 2**.

1. Methylprednisolone (100 mg) IV + antihistamine IV or PO.
2. Imlifidase 0.25 mg/kg body weight over 15 min (0.2 µm filter—caveat: this is not a standard filter) i.v.
3. Four hours after imlifidase: negative CDC/FC-XM—at least against T lymphocytes or unseparated cells with DTT → implantation.
4. Four hours after imlifidase: positive CDC/FC-XM → second dose of imlifidase (0.25 mg/kg body weight, within ≤24 h after initial administration) → CDC/FC-XM again 2 h later.
5. If CDC/FC-XM remains positive → no transplantation is possible.

We recommend reading the imlifidase product information before administration and taking it into account.

### Crossmatch conversion

In accordance with the imlifidase approval text, confirmation of crossmatch conversion should be performed using a SAB assay from undiluted serum, taken no earlier than 3 months prior to imlifidase administration and, as a rule, within 4 h of administration. A CDC-XM with isolated T lymphocytes or unseparated cells, and optionally isolated B lymphocytes, must be performed prospectively after imlifidase administration and must be negative before transplantation can take place (see the German Medical Authorities’ guidelines on recipient protection and kidney transplantation). Additionally, a flow cytometric crossmatch (FC-XM) with T and/or B lymphocytes can be used either prospectively or retrospectively.

### Immunosuppression

Induction (days 0–10).

- Methylprednisolone day 0: 500 mg, days 1–3: 250 mg, day 4: 125 mg.
- Tacrolimus (Tac), ≤8 h post-transplant (trough level 10–12 ng/mL). The initial dose should be administered prior to transplantation (dosage according to product information).
- Mycophenolate (MMF 2 × 1,000 mg or MPA 2 × 720 mg). The initial dose should be administered prior to transplantation (dosage according to product information).
- rATG 1.5 mg/kg body weight or ATLG 3 mg/kg body weight on days 4–7 (alternatively eATG 40 mg/kg body weight on days 0–4, off-label).
- If necessary, B-cell-depleting therapy, e.g., rituximab 375 mg/m² body surface area on day 7 (off-label).
- Usually IVIG 2 g/kg body weight on days 9–10 (off-label).

The use of anti-CD38 antibodies, such as daratumumab (off-label), on day 7 to deplete NK cells and modulate activated T, B, and plasma cells for the prevention of AMR can currently only be based on theoretical considerations and individual case reports due to a lack of data and should not be regarded as an established standard for AMR prevention after imlifidase-enabled transplantation (22). Daratumumab has been used off-label as a plasma-cell- and NK-cell-targeting strategy in selected cases for desensitization and treatment of AMR, but current evidence is limited to mechanistic rationale, case reports and small series (41–46). Its use should therefore be restricted to individualized MDT decisions, preferably within a clinical trial or registry. Daratumumab should not be considered an alternative B-cell-depleting therapy to rituximab, because its principal targets are CD38-expressing plasma cells and NK cells rather than CD20-positive B cells. In this context, daratumumab can be used either in addition to rituximab or as an alternative B-cell-targeted therapy instead of rituximab. Neither of the therapies should be interpreted as a mandatory protocol component in this rapidly evolving field. Felzartamab, another anti-CD38 monoclonal antibody, has shown proof of concept in a randomized phase 2 trial in late AMR and is currently being evaluated in phase 3 (47). However, felzartamab remains investigational and is not approved for AMR treatment. Regulatory designations, including orphan drug designation, should be interpreted as development incentives and not as evidence of an established clinical standard of care.

### Maintenance therapy (≥month 2)

Tacrolimus 8–10 ng/mL, mycophenolate mofetil 2 g/day, prednisolone 5 mg/day.

*Option*: Pre-rituximab (−14 days) for planned living kidney donation (in which case imlifidase is also off-label). In living donors, 1,800 mg of daratumumab can also be administered for immunomodulation and depletion of NK cells a few days before the planned transplant (22). The French recommendation is for IVIGs on days 4 and 5 post-TX (19).

### Infection prophylaxis

**Table 3** provides an overview of the recommended infection prophylaxis measures.

**Table 3:** Infection prophylaxis and recommendations for vaccinations when using imlifidase.

Time period	Measure	Comment
Day 0–4 weeks after KTX	Oral antibiotics for respiratory tract infections	E.g., amoxicillin/clavulanic acid, azithromycin
6–12 months after KTX	Pneumocystis prophylaxis	Cotrimoxazole, pentamidine if necessary
0–6 months	CMV prophylaxis	Risk-adjusted
In patients at risk	*Candida* prophylaxis	Fluconazol
Before KTX	Vaccinations according to German national (STIKO) recommendations, including pneumococcal and meningococcal vaccines	Complete before therapy or admission to a desensitization program

- Complete vaccination protection prior to listing or in cases of planned desensitization with imlifidase according to the current German national (STIKO) recommendation, including pneumococci and meningococci in cases of potential C5 blockade.
- In addition to standard prophylaxis, a 4-week course of antibiotic prophylaxis with oral preparations that typically cover respiratory pathogens, e.g., amoxicillin/clavulanic acid or azithromycin, should be administered after imlifidase administration.
- Trimethoprim-SMX for ≥6–12 months or pentamidine inhalation in case of trimethoprim-SMX allergy.
- Valganciclovir or letermovir for 3–6 months in accordance with the consensus recommendations of the Transplantation Society (48) or the German AWMF guideline (https://register.awmf.org/de/leitlinien/detail/093-002).

### DSA monitoring after transplantation

Post-transplant DSA monitoring serves to detect DSA rebound and initiate appropriate rejection therapies at an early stage (49).

The following procedure is recommended:

- Day 0 (transplant day): The preoperative serum before imlifidase documents the initial immunological status. The serum after imlifidase forms the reference point for the success of desensitization and further post-transplant DSA monitoring.
- At least days 3, 5, 7, 10, 14, and 21: Check DSA MFI to detect rebound and further dynamics, as rebound may have prognostic significance and new treatment options for AMR are available with anti-CD38 antibodies (46, 47, 50).
- At least months 1, 3, 6, and 9: Evaluate DSA trends in terms of persistence or decline.
- Annual monitoring to detect long-term trends.
- Additionally, indication-driven DSA monitoring and graft biopsies should be considered.
- If necessary, serum should be stored at −20°C for later analysis.

Preliminary analyses of the unpublished ISKIA registry show a close association between post-transplant DSA-MFI and total DSA-MFI with AMR incidence. These data support the monitoring of DSA in the early stages and the interpretation of trends.

In cases of DSA positivity following transplantation, determining donor-specific circulating deoxyribonucleic acid (dd-cfDNA) every 2 weeks in the first quarter is also recommended to better assess kidney damage and diagnose subclinical AMR promptly (see (51, 52)).

Because early DSA rebound is expected after imlifidase-enabled transplantation, an early protocol biopsy should be considered between days 7 and 10 after transplantation, where clinically feasible, to detect early subclinical AMR during the rebound window. If biopsy at days 7–10 is not feasible because of bleeding risk, anticoagulation, delayed clinical stabilization or local logistics, biopsy at day 14 or day 21 should be discussed, as early detection of AMR will provide a rationale for early treatment (46, 53). Additional protocol biopsies should be performed at months 3 and 12 to detect or rule out persistent or late subclinical AMR. Indications for biopsies should be performed at any time in cases of graft dysfunction, relevant DSA rebound, dd-cfDNA elevation, or other clinical suspicion of rejection. Additional molecular analyses, such as MMDx, can be helpful for diagnosis, especially in unclear cases (54).

### Management of AMR

As a general rule, basic immunosuppression and adherence should be reviewed and, if necessary, optimized. The following recommendations are based largely on the expert consensus of Böhmig et al. (53).

#### Early AMR (<6 months)

##### Steroid pulse therapy

Plasmapheresis/immunoadsorption daily for 3 days, then every 2–3 days until day 14.

+ IVIG 2 g kg^-^¹.

AT(L)G in cases of concomitant T-cell rejection (TCMR).

C5 inhibition (off-label or investigational) in thrombotic microangiopathy (TMA).

Anti-CD38 antibody (off-label or investigational) in severe microvascular inflammation (MVI).

#### Late, chronically active AMR (>6 months)

Optimized basic immunosuppression + anti-CD38 antibody (off-label or investigational), e.g., daratumumab 16 mg kg^–1^ weekly for 4 weeks, then monthly for 3–6 months, whereby the determination of dd-cfDNA can support therapy management.

### Living kidney donation transplantation

Following provisional approval by the EMA, imlifidase is reserved for use in kidney transplantation in a postmortem setting. Nevertheless, HLA-incompatible living kidney transplantation can also benefit from crossmatch conversion using imlifidase. However, patients must clarify the coverage of imlifidase costs in the off-label setting of living kidney transplantation with their insurance providers in advance.

### Registration requirements and quality assurance

Patients who have been treated with or without imlifidase after being delisted from NAHAs are recommended to be included in a registry or clinical trial. Prospective registry data and study data are crucial for validly demonstrating the efficacy, safety, and cost-effectiveness of the imlifidase strategy in Germany.

## Discussion

The DTG and DGI professional associations have made recommendations on controlled delisting of unacceptable antigens, supported by conventional desensitization regimens with intensified immunosuppression post-transplant, and/or by the use of imlifidase, which are outlined here (see **Figure 2** for a synopsis). **Table 1** contextualizes these approaches alongside compatibility-preserving strategies, including acceptable mismatch allocation, compatible living-donor pathways and kidney paired donation, and potential incompatible living donation. The aim is to improve access to kidney transplantation for HS patients. Without such innovative strategies, a significant proportion of this patient group will remain on the waiting list long term and experience significantly increased mortality while on dialysis (2–4). .

The IgG endopeptidase imlifidase can rapidly reverse positive crossmatch results by cleaving circulating IgG antibodies within 60–240 min. Phase II data (HIGHDES) show that crossmatch conversion occurs in 89% of cases, with 3-year patient and graft survival rates of 84% and 78%, respectively (16, 18). Five-year data from 46 imlifidase-assisted kidney transplants in Sweden, France, and the USA, confirm patient survival rates of 90% and death-adjusted graft survival rates of 82% (17). These results are comparable to those of conventional incompatibility protocols, but lead to significantly shorter waiting times. At the same time, clinical AMR can be expected in approximately 40% of patients.

In parallel, delisting strategies reduce vPRA by gradually removing non- or low-complement-binding HLAabs. A Spanish multicenter cohort study involving 48 patients reduced patient’s cPRA from 100% to 98.3% and achieved a transplant rate of 63% within 3 months. Of these patients, 18 were transplanted without imlifidase being used, provided that FC/CDC-XM remained negative (12). However, the 1-year AMR rate in these 18 patients was 43.7%, particularly in cases involving DSA against HLA-A in combination with HLA-DRB1/-DQB1.

Due to the high AMR rate associated with the proposed desensitization approach involving imlifidase, any long-term successful strategy must also consider new therapeutic options for preventing and treating AMR (46, 47, 53). The most effective approach would be to use concomitant therapy to completely prevent AMRs. Initially, successful approaches were observed in living donors where NK effector cells were depleted, and immunomodulation with an anti-CD38 antibody was performed in advance (22). For logistical reasons, direct preoperative administration of an anti-CD38 antibody is impractical, particularly since the antibody would be rapidly cleaved by imlifidase. However, administration at the end of the first week would be feasible, particularly in cases of early DSA rebound. Further clinical studies are needed to test such hypotheses and further develop the protocols. The same applies to the treatment of early fulminant AMR, where previous treatment concepts have not demonstrated convincing success rates (43–45, 53, 55, 56). Although data on the successful treatment of AMR with anti-CD38 antibodies are growing, these case reports and series tend to refer to slowly progressive chronic active AMR. Nevertheless, the effective depletion of NK cells in the kidney following anti-CD38 antibody therapy (47, 57) could potentially be an effective treatment for early severe rejection.

Overall, desensitization (conventional or with imlifidase) and the delisting of unacceptables are complementary approaches. While desensitization reduces waiting times in cases of positive crossmatching, delisting unacceptables enables patients with low to moderate non-complement-binding DSAs to undergo transplantation. Both procedures require multidisciplinary expertise and robust laboratory monitoring (DSA-MFI, complement binding, and epitope analysis). We recommend systematically recording cases in a German registry.

We therefore recommend the following:

- The gradual delisting of unacceptables in cases involving low donor frequency, long waiting times, severe dialysis-related issues, and negative T-cell CDC. This is to ensure compliance with the principles of urgency and chances of success in individual cases (see also the German recommendations on unacceptables (11)).
- Use imlifidase or other suitable desensitization strategies in experienced centers for suitable patients with positive vXM.
- Continuous data collection in a German desensitization registry (DeSiRe), alongside prospective monitoring of AMR rates and long-term outcomes.

### Comments/limitations

The MFI limits referenced in this recommendation refer exclusively to SAB assays from One Lambda (West Hills, CA, USA). These limits can be applied to SAB assays from Werfen (Barcelona, Spain) using the publication by Karahan et al. as a rough guide (58). There is currently little comparative data available for the single antigen spot microarray (BAG Diagnostics, Lich, Germany) (59–61); a further publication is in preparation.

## Conclusion

In conclusion, an individualized, data-driven approach is crucial to finding the optimal balance between the chance of transplantation and the risk of rejection on a case-by-case basis. Combining risk-stratified delisting of unacceptables with desensitization for XM-positive situations provides an evidence-informed, expert-consensus-based framework for selected HS patients in Germany who have no realistic compatible transplant option or in whom further waiting has become a detrimental clinical option. Because comparative evidence remains limited, this pathway should not be interpreted as a universal standard of care in a rapidly evolving field, but as a structured strategy for experienced centers with prospective registry-based outcome assessment. It is crucial to systematically capture the outcomes in order to move the field from expert consensus to an evidence-based approach.
